# Chemical Modification of Acrylonitrile-Divinylbenzene Polymer Supports with Aminophosphonate Groups and Their Antibacterial Activity Testing

**DOI:** 10.3390/molecules29246054

**Published:** 2024-12-23

**Authors:** Ileana Nichita, Lavinia Lupa, Aurelia Visa, Ecaterina-Stela Dragan, Maria Valentina Dinu, Adriana Popa

**Affiliations:** 1 Faculty of Veterinary Medicine, University of Life Science “King Mihai I”, 119 Calea Aradului, 300465 Timisoara, Romania; ileananichita@usab-tm.ro; 2 Faculty of Chemical Engineering, Biotechnology and Environmental Protection, Politehnica University Timișoara, 6 Vasile Parvan Blvd., 300223 Timisoara, Romania; lavinia.lupa@upt.ro; 3 “Coriolan Drăgulescu” Institute of Chemistry, 24 Mihai Viteazul Blv., 300223 Timisoara, Romania; avisa@acad-icht.tm.edu.ro; 4 “Petru Poni” Institute of Macromolecular Chemistry, 41A Aleea Grigore Ghica Vodă, 700487 Iași, Romania; dinu.valentina@icmpp.ro

**Keywords:** acrylonitrile-divinylbenzene, aminophosphonate, multicomponent reactions, antimicrobial materials, Weibull model

## Abstract

Bacterial contamination is a major public health concern on a global scale. Treatment resistance in bacterial infections is becoming a significant problem that requires solutions. We were interested in obtaining new polymeric functionalized compounds with antibacterial properties. Three components (polymeric amine, aldehyde, and phosphite) were used in the paper in a modified “one-pot” Kabachnik–Fields reaction, in tetrahydrofuran at 60 °C, to create the N-C-P skeleton in aminophosphonate groups. Two copolymers were thus prepared starting from an acrylonitriledivinylbenzene (AN-15%DVB) copolymer containing pendant primary amine groups modified by grafting aminophosphonate groups, i.e., aminobenzylphosphonate (Bz-DVB-AN) and aminoethylphosphonate (Et-DVB-AN). The two copolymers were characterized by FT-IR spectroscopy, SEM-EDX, TGA, and antibacterial properties. It was shown that the novel products have antibacterial qualities against *S. aureus* and *E. coli* bacteria. The sample with the strongest antibacterial activity was Et-DVB-AN. We assessed how well the Weibull model and the first-order kinetic model represent the inactivation of microbial cells in our samples. The main advantage of the new antibacterial agents developed in this work is their easy recovery, which helps to avoid environmental contamination.

## 1. Introduction

Various antimicrobial polymers have been designed in recent decades owing to the antimicrobial resistance developed by pathogens associated with the overuse of the conventional antimicrobial drugs. Among them, polysaccharides such as chitosan modified by attaching functional moieties, which enhances their intrinsic antimicrobial properties [1,2,3,4], and some synthetic polymers [4,5,6,7,8], have been lately reported. The main interest has been focused on identifying the most effective functional groups that provided the optimal antimicrobial activity. Multicomponent reactions (MCRs), which introduce several functional groups into a single one-pot process, are powerful tools for synthesizing a variety of important chemicals, making the production of multifunctional molecules more accessible. The chemistry of MCRs has been widely explored, as the majority of atoms from three or more compounds in an MCR are efficiently integrated into a single product [9]. The first MCR was performed by Strecker in 1850 [10], aiming to synthesize α-amino cyanide. Since then, numerous MCRs have been discovered, including the Biginelli, Gewald, Hantzsch, Kabachnik–Fields (KF), Mannich, Passerini, and Ugi reactions [11]. Among these, the KF three-component reaction plays a particularly important role. Developed in 1952 [12], the KF reaction remains the most efficient method for the formation of carbon–phosphorus bonds, utilizing three common starting materials—an amine, an aldehyde/ketone, and a dialkylphosphonate—to produce α-aminophosphonates [1,13]. Phosphorus-containing materials have garnered increasing attention due to their versatility across a range of applications. They have been used worldwide as water treatment additives, agricultural fertilizers, anticorrosive surface inhibitors, flame retardants, and pharmaceuticals [14,15,16]. Our previous works detailed a one-pot KF MCR method for grafting aminophosphonic acid and aminophosphinic acid groups onto styrene-divinylbenzene (DVB) copolymers, with 15%, 6.7%, or 1% DVB content [17,18,19]. Aminophosphonates, as structural analogs of amino acids (“bioisosterism”) have attracted special attention because of their notable biological activities. Bioisosteres are chemical substituents or groups with similar physical or chemical properties that can mimic biological effects [20,21]. In the case of aminophosphonates, their bioisosterism involves the substitution of -OH and -NH groups. Phosphorus, in the form of organic phosphates, also plays a crucial role in biochemical processes.

Through polymer-analogous processes, we have previously developed styrene-DVB copolymers functionalized with pendant groups (e.g., α-hydroxyphosphonic acids, aminophosphonic groups, aminophosphinic groups) [17,18,19]. In our previous studies, insoluble polymers were tested as antibacterial agents and different results were obtained, depending on their structure. For example, the study carried out on two products, aminobenzylphosphonic acid (BzAmPhonic) and aminopropylphosphonic acid (PrAmPhonic), grafted on copolymer (S-6.7% DVB), showed good antimicrobial activity, but the best antibacterial activity was obtained with the PrAmPhonic sample for both *P. aeruginosa* and *S. aureus* [19]. For other compounds with aminophosphinic acid groups grafted onto styrene-6.7%divinylbenzene copolymer and α-hydroxyphosphonic acid groups, it was observed that the bioactive groups have an antibacterial effect against *S. aureus*, *E. coli*, *P. aeruginosa*, and *B. cereus* [17,18].

Crosslinked copolymers of acrylonitrile (AN) with difunctional monomers such as DVB or ethyleneglycol dimethacrylate (EGDMA) have been synthesized either as beads [22] or as composites with mesoporous silica [23,24], owing to the high versatility of nitrile groups for creating a wide variety of functional materials. Thus, novel chelating resins bearing iminodiacetate groups derived from porous AN-DVB copolymers have been developed and investigated for their ability to remove Cu(II), Co(II), and Ni(II) from aqueous solutions [22]. Composite sorbents consisting of ion exchangers bearing amidoxime functional groups, derived from AN-EGDMA copolymers embedded into the mesoporous silica pores, have been successfully used to adsorb strontium and cesium [23], or arsenic [24], from aqueous solutions.

In this work, two porous AN-15%DVB copolymers were synthesized using either 2-ethyl-1-hexanol (2-EH) (code SV3) or toluene (code SV4) as porogen agents, and after functionalization with primary amine groups, they served as starting materials for the development of novel copolymers with antibacterial properties. These properties were achieved by modifying the amine moieties into aminophosphonate groups using the KF strategy. The antibacterial properties of new products against *E. coli* and *S. aureus* were demonstrated. The sample Et-DVB-AN has the strongest antibacterial activity. The ability to be readily recovered to prevent environmental contamination is the primary benefit of the novel antibacterial agents created in this work.

## 2. Results and Discussion

**Scheme 1:**
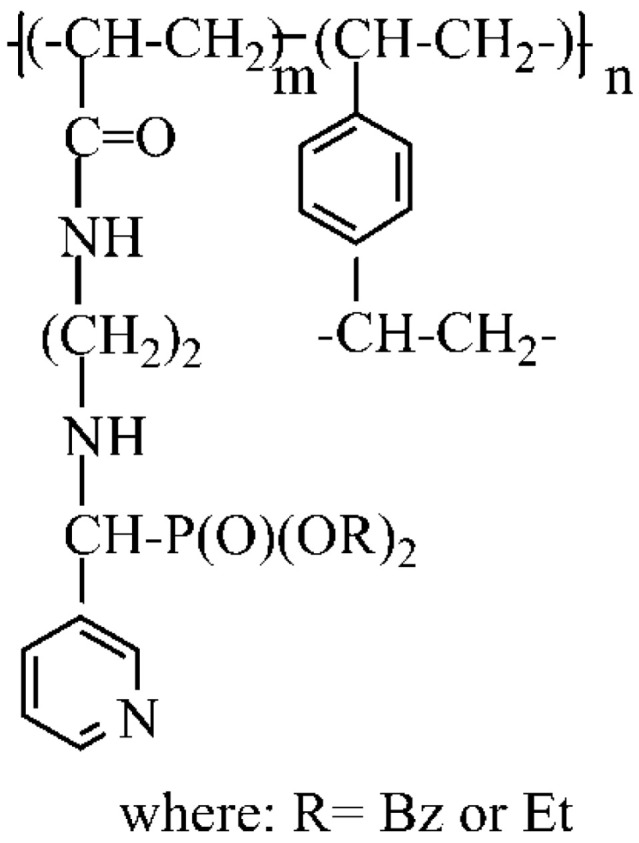
The structure of the functionalized copolymers: Bz-DVB-AN and Et-DVB-AN.

The structures of the final products, Bz-DVB-AN and Et-DVB-AN, are presented in Scheme 1. The proposed structure resulted from the functionalization of the copolymers SV3 and SV4 with 3-pyridinecarboxyaldehyde, and either dibenzylphosphite (Bz-DVB-AN) or diethylphosphite (Et-DVB-AN).

### 2.1. Chemical and Morphological Characterization of Bz-DVB-AN Copolymer

**Figure 1:**
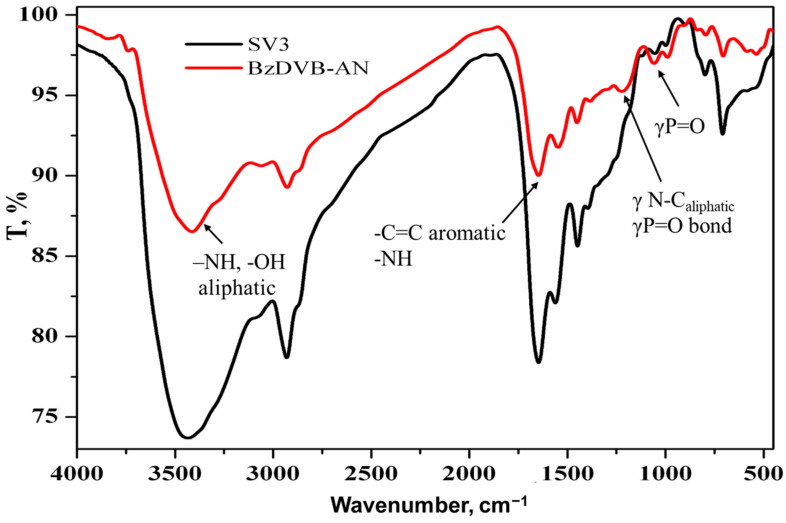
FTIR spectra of SV3 copolymer and Bz-DVB-AN sample.

The following bands can be seen in the SV3 pristine copolymer’s FTIR spectra (Figure 1): N-H groups were allocated to the primary amine at 1448 cm^−1^ and 708 cm^−1^; 3434 cm^−1^ and 1647 cm^−1^ were ascribed to the -NH_2_ and -CH_2_ bonds at 2931 cm^−1^. The FTIR spectrum of the Bz-DVB-AN sample showed adsorption bands in the following areas: 3510–3350 cm^−1^ attributable to (aliphatic–NH, and -OH), 1680–1570 cm^−1^ attributable to (-C=C, aromatic ring, NH in secondary and primary amines, respectively), 1360–1210 cm^−1^ attributed to (γN-Caliphatic, γP=O bond), 1169–950 cm^−1^ attributed to (γP=O) and 880–709 cm^−1^ assigned to (N-H groups in the amine primary and P-OR) [18,19].

**Figure 2:**
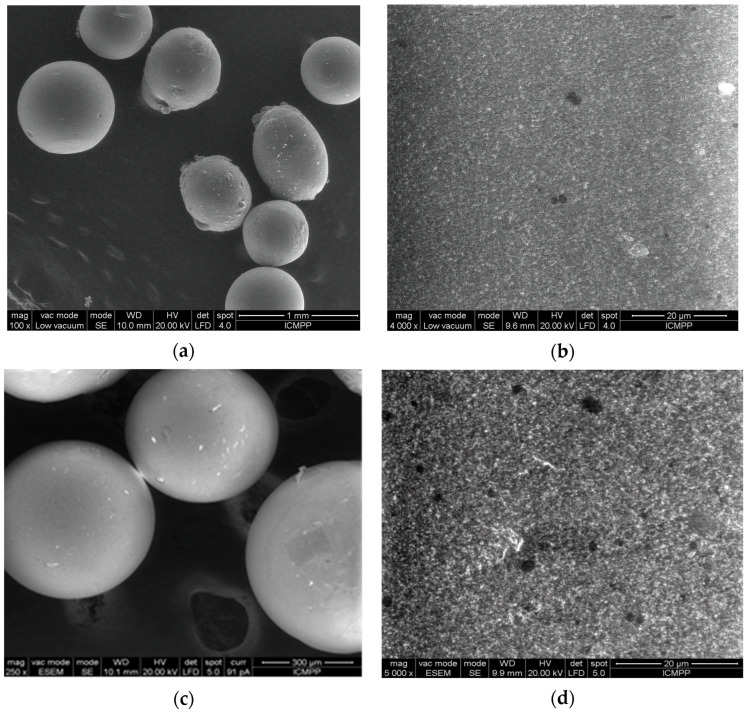
SEM images of SV3 (**a**,**b**) and Bz-DVB-AN (**c**,**d**) copolymers; mag: 100× (**a**), 4000× (**b**), 250× (**c**) and 5000× (**d**).

SEM images of the pristine SV3 copolymer and of the aminophosphonate Bz-DVB-AN copolymer are presented in Figure 2. It can be observed that both before and after the K-F reaction, the copolymer has a uniform surface morphology.

**Figure 3:**
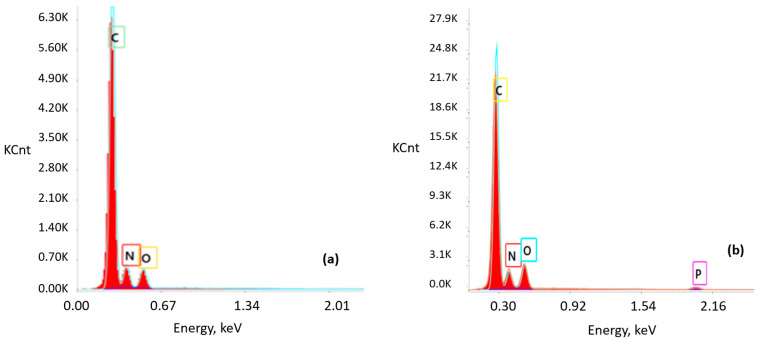
EDX spectra of (**a**) SV3 and (**b**) Bz-DVB-AN copolymers.

The EDX spectrum of Bz-DVB-AN copolymer, presented in Figure 3b, indicates that the functionalization of SV3 copolymer with 3-pyridinecarboxyaldehyde and dibenzylphosphite occurred.

The values of elemental analysis and those obtained from the semi-quantitative EDX analysis are presented in Table 1. When the EDX for SV3 and the product Bz-DVB-AN are compared, the copolymer is functionalized with the dibenzylphosphonate group, as evidenced by the decrease in N% from 21.7% for SV3 to 18% for the final product and an increase in O% from 10% for SV3 to 13.7% for Bz-DVB-AN.

**Figure 4:**
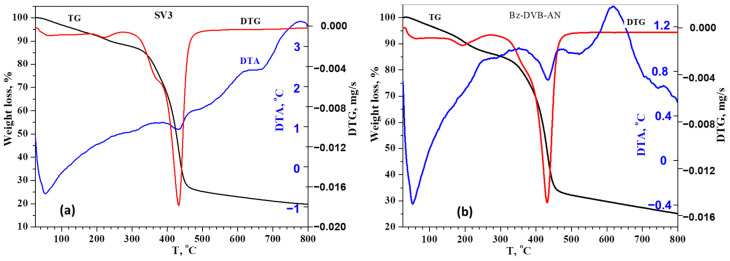
Thermogravimetric analysis of the pristine SV3 copolymer (**a**) and Bz-DVB-AN (**b**).

The findings of the TG-DTG-DTA analysis of SV3 in nitrogen are displayed in Figure 4a. The mass loss occurred in three stages: the first stage, which was 6.7% (~59.9 °C) as a result of physically adsorbed water evaporating; the second stage, which was 4.7% (~223.2 °C) as a result of pendant group degradation; and the third stage, which was 64.8% (~433.4 °C) as a result of DVB-AN degradation. A total of 19.8% of the residue remained after 80.2% of the total mass was eliminated at 800 °C. Figure 4b shows the TG-DTG-DTA analysis of Bz-DVB-AN in nitrogen. It is evident that the mass losses occurred in three phases. The evaporation of physically adsorbed water was responsible for the first stage (25–140 °C) with—3.56% (~58.6 °C); the pendant group degradation for the second stage (140–360 °C) with—10.21% (~193 °C), and the DVB-AN degradation for the third stage (360–660 °C) with—61.12% (~432.1 °C). A total of 74.89% of the whole mass was lost, leaving 25.11% of residue at 800 °C. Good thermal stability is demonstrated by the functionalized copolymer with the aminophosphonate group (Bz-DVB-AN).

### 2.2. Chemical and Morphological Characterization of Et-DVB-AN Sample

**Figure 5:**
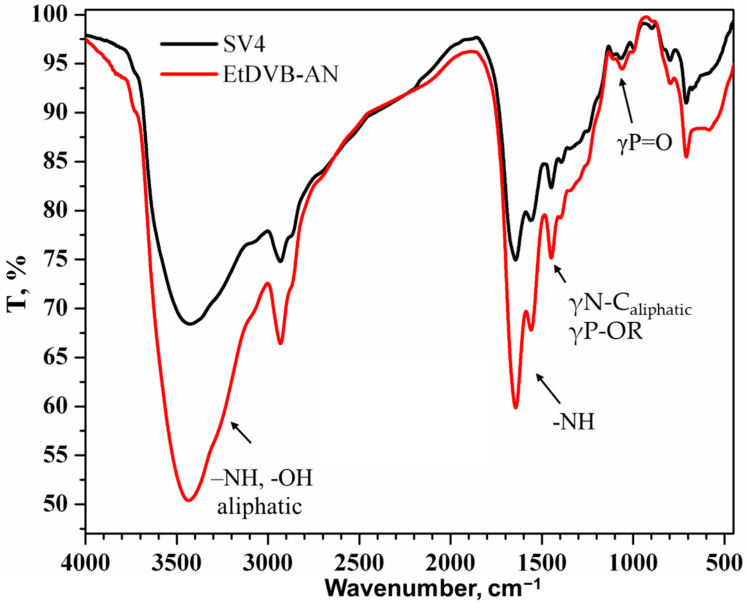
FTIR spectra of SV4 copolymer and Et-DVB-AN sample.

The FTIR spectra of the SV4 pure copolymer show the following bands (Figure 5). The bands found at 1448 cm^−1^ and 709 cm−1 were assigned to N-H groups, while the bands situated at 3429 cm^−1^ and 1643 cm^−1^ were assigned to -NH_2_ groups. The Et-DVB-AN sample’s FTIR spectrum in Figure 5 displayed notable adsorption bands in the following areas: 3545–3350 cm^−1^ attributed to (-OH and aliphatic-NH groups), 1660–1569 cm^−1^ attributed to (NH in secondary and primary amines, respectively), 1460–1390 cm^−1^ assigned to (γN-C-aliphatic, γP-OR), 1160–990 cm^−1^ assigned to (γP=O bond), and 800–690 cm^−1^ assigned to (N-H groups in the amine primary and P-OR) [18,19].

**Figure 6:**
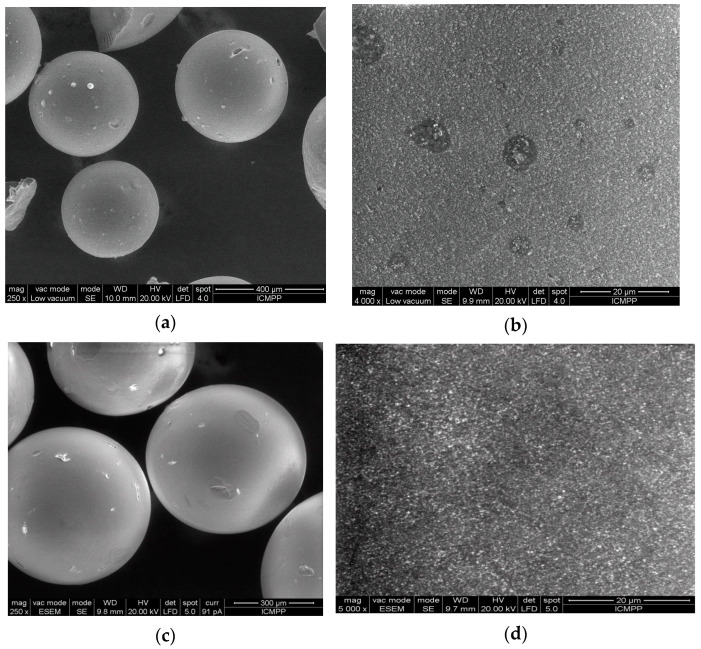
SEM images of SV4 (**a**,**b**) and Et-DVB-AN (**c**,**d**) copolymers; mag: 250× (**a**), 4000× (**b**), 250× (**c**) and 5000× (**d**).

SEM images of the pristine SV4 copolymer and of the aminophosphonate Et-DVB-AN copolymer are presented in Figure 6. It can be seen that both the pristine copolymer SV4 and the functionalized copolymer have a homogeneous surface morphology.

**Figure 7:**
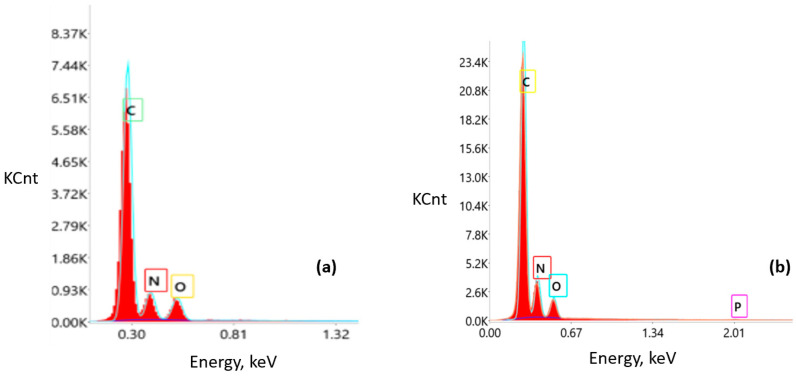
EDX spectra of SV4 (**a**) and Et-DVB-AN (**b**).

EDX spectra presented in Figure 7 show the result of functionalizing acrylonitrile-15%divinylbenzene copolymers (SV4) using diethylphosphite and 3-pyridine carboxyaldehyde, respectively.

The values of elemental analysis and those obtained from the semi-quantitative EDX analysis and elemental analysis are presented in Table 2.

**Figure 8:**
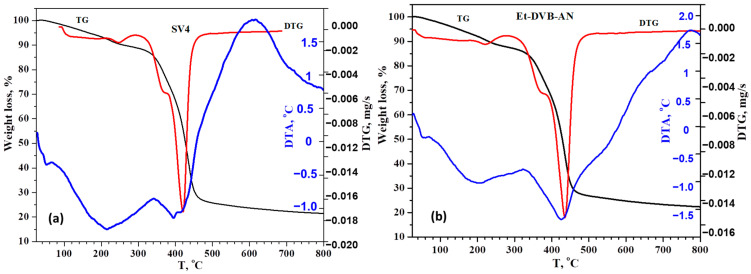
Thermogravimetric analysis of the pristine SV4 copolymer (**a**) and Et-DVB-AN (**b**).

The findings of the TG-DTG-DTA analysis of SV4 in nitrogen are displayed in Figure 8a. The mass loss of physically adsorbed water evaporating in the first stage (25–120 °C) was 5.6% (~60 °C); in the second stage (120–340 °C), it was 4.9% (~220.2 °C) due to pendant group degradation; and in the third stage (340–660 °C), it was 68.0% (~434.5 °C) due to DVB-AN degradation. A total of 21.5% of the residue remained after 78.5% of the total mass was eliminated at 800 °C. Figure 8b displays the TG-DTG-DTA study for the Et-DVB-AN sample. The synthesis product Et-DVB-AN was studied in nitrogen, and three steps of mass losses were noted: the evaporation of the physically adsorbed water was responsible for the first stage (25–140 °C), with—6.69% (~55.9 °C); the pendant group degradation was responsible for the second stage (140–360 °C), with—5.61% (~220.2 °C); and the DVB-AN degradation was responsible for the third stage (360–660 °C), with—65.37% (~422 °C). At 800 °C, 22.33% of the residue was still present after a total mass loss of 77.67%. Thus, the copolymer functionalized with the aminophosphonate group Et-DVB-AN shows a good thermal stability.

Comparing the two samples of copolymers functionalized with aminophosphonate groups (Bz-DVB-AN and Et-DVB-AN), better stability was observed in the case of the Bz-DVB-AN sample than for Et-DVB-AN.

### 2.3. Antibacterial Study

**Figure 9:**
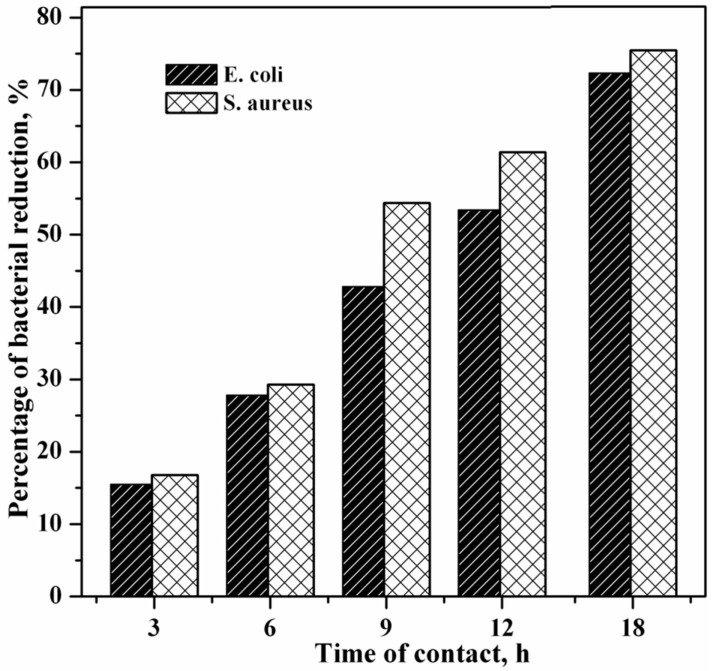
Percentage of bacterial reduction induced by the Bz-DVB-AN sample at different intervals of time.

**Figure 10:**
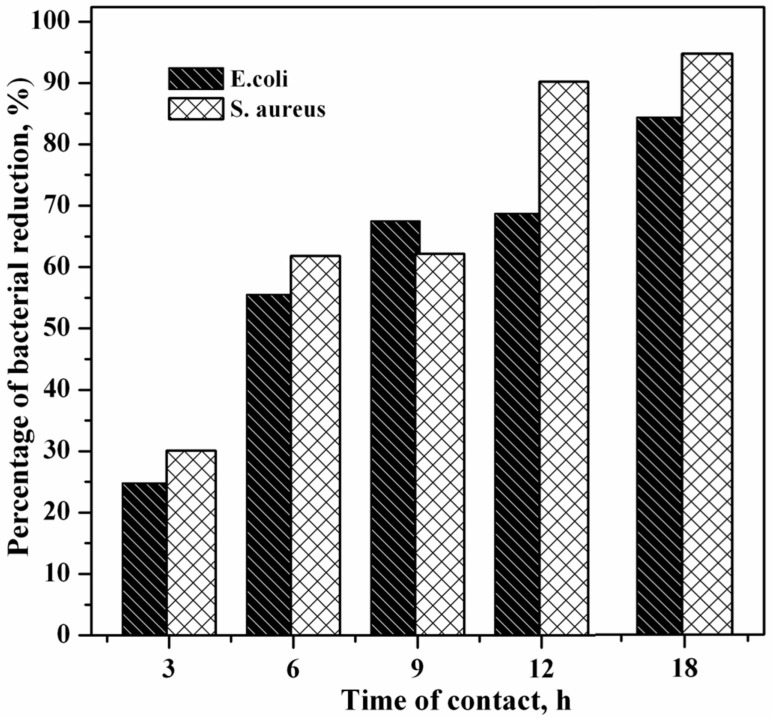
Percentage of bacterial reduction induced by the Et-DVB-AN sample at different intervals of time.

The characterized material was subject to antibacterial tests using *E. coli* and *S. aureus* and the colony forming unit assay method for different time contacts. The variation regarding the number of total germs (CFU/mL) and percentage of bacterial reduction (P, %) at different contact times with the two antimicrobial materials are presented in Table S1 in Supplementary Materials. The number of viable cells from each bacteria type was significantly reduced by the studied materials and the inactivation effectiveness was dependent on the treatment time. Both materials, after 18 h of contact with the bacterial suspensions, showed the microbial reduction presented in Table S1. In the case of the action on *E coli*, the percentage reduction in the Bz-DVB-AN substance was 72.2, but the Et-DVBAN substance had a percentage reduction of 84.4. In the case of the *S. aureus* species, the percentage reduction of the Bz-DVB-AN substance was 75.4, and that of the Et-DVBAN substance was 94.8. The effectiveness of microbial reduction by the studied materials is presented in Figure 9 and Figure 10. The materials exhibit antibacterial activity three times higher than that of the standard sample within the first three hours of contact. Both materials are more effective against *S. aureus* compared to *E. coli*. The Et-DVB-AN material demonstrates 20% better efficacy than the Bz-DVB-AN material to *E. coli*. The higher effectiveness in Gram-positive bacteria may be explained by the electrostatic interaction between the electronegative charge of the sample Et-DVB-AN and the positive charges on their walls. The reduction of more than 50% of the Gram-positive bacterial load was manifested after 9 h of contact; this point was observed for the Gram-negative ones only after 12 h of contact. In *S. aureus*, the effect of the sample Et-DVB-AN is expressed at a bacteriostatic (94.8%) to bactericidal (99%) efficiency (see Figure 9 and Figure 10).

The results regarding the logarithmic survival fraction versus contact time for both pathogens in the case of the two studied materials are presented in Table 3.

To efficiently optimize treatment settings, a solid mathematical model reflecting the kinetics of microbial cell inactivation must be discussed. In this case, two mathematical models were applied for kinetic inactivation modeling of *E. coli* and *S. aureus* onto Bz-DVB-AN and Et-DVB-AN: the first-order kinetic model and Weibull model. The first-order kinetic model is a commonly used predictive tool for microorganism inactivation patterns. It is based on the assumption that the microbial population has a similar level of resistance and that there is a linear relationship between treatment time and the number of surviving cells [25,26].

The first-order kinetic model is presented in Equation (1).

(1) $$log\frac{N_{t}}{N_{0}}=-\frac{t}{D_{T}}$$

where the starting and surviving populations of bacteria following treatment time *t* (h) are denoted by *N*_0_ and *N_t_* (CFU/mL), respectively. The decimal reduction time, or *D_T_* (h), represents the amount of time needed to inactivate 90% of the bacterial population.

**Figure 11:**
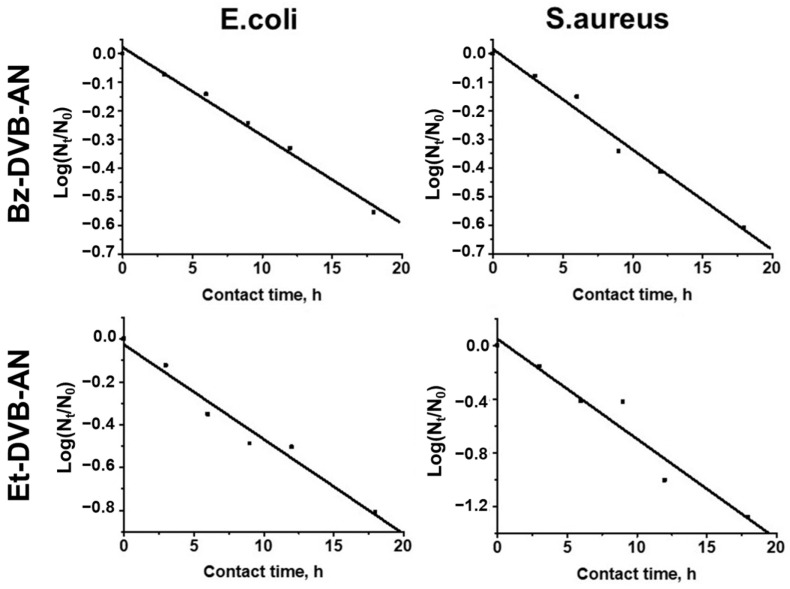
First-order kinetic model of *E. coli* and *S. aureus* reduction by treatment with Bz-DVB-AN and Et-DVB-AN.

The linear representations of the first-order kinetic model of *E. coli* and *S. aureus* reduction by treatment with Bz-DVB-AN and Et-DVB-AN are presented in Figure 11. The decimal reduction time obtained in each case is presented in Table 4.

It could be observed that the first-order kinetic model could be accurately applied for the experimental data fitting due to the high obtained values for R^2^. Table 4 showed that the D_T_ values for Bz-DVB-AN and Et-DVB-AN were 32.4 and 28.4 for *E. coli* and 22.6 and 13.4 for *S. aureus*. These findings suggest that Et-DVB-AN is more effective against both pathogens, as it achieved a greater reduction in microbial counts within shorter treatment periods. This observation aligns with the improved inactivation results seen in the experimental data, indicating that the material’s structure affects its efficiency. Additionally, the decimal reduction in *S. aureus* for both materials showed a better effectiveness, indicating that the dynamics of bacterial inactivation differ between species.

The first-order kinetic model is typically only applicable to log-linear inactivation patterns. Since microbial inactivation curves frequently show non-log-linear relationships, the Weibull model is typically used to analyze non-log-linear survival curves and is describe by Equation (2) [25,26,27,28].

(2) $$log\frac{N_{t}}{N_{0}}=-{bt}^{n}$$

where *n* is the dimensionless shape parameter and *b* is the distinctive slope or scale parameter (min^−1^).

**Figure 12:**
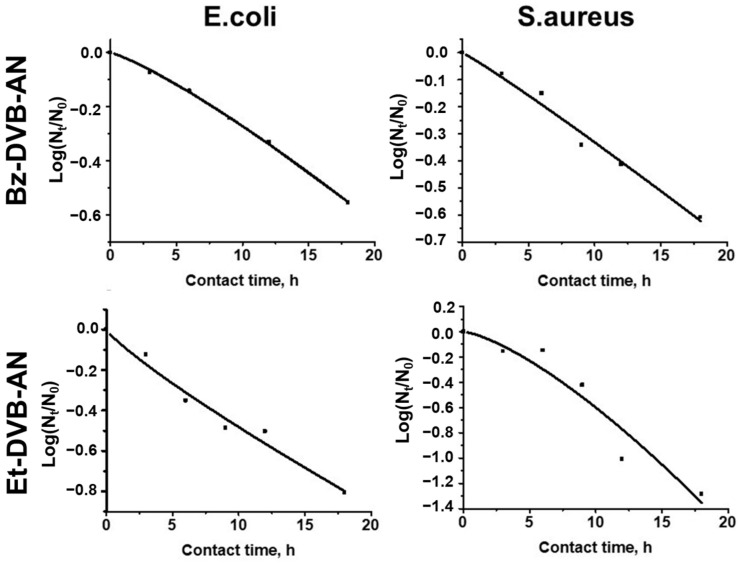
Weibull model of *E. coli* and *S. aureus* reduction by treatment with Bz-DVB-AN and Et-DVB-AN.

Inactivation resistance can be interpreted using concavity; downward concavity (*n* > 1) indicates shoulder or decreasing resistance, while upward concavity (*n* < 1) indicates tailing or growing resistance [27]. Therefore, variations in the resistance of the bacteria to the treatment are shown by the Weibull inactivation curve. The Weibull model representations of *E. coli* and *S. aureus* reduction by treatment with Bz-DVB-AN and Et-DVB-AN samples are presented in Figure 12, and the obtained kinetic parameters are summarized in Table 5.

The Weibull model suggests a good fit of the experimental data obtained in all cases, with chi-square values less than 0.003. It could be observed that the obtained graphics present a downward concave curve (*n* > 1) for *E. coli* and *S. aureus* pathogens treated with Bz-DVB-AN and for *S. aureus* on Et-DVB-AN. This indicates that the *S. aureus* bacteria are more vulnerable to the materials under study. In the case of *E. coli* treated with Et-DVB-AN, the fitted model presents an upward concavity (*n* < 1), assuming that the less-sensitive microorganisms were deactivated at a relatively fast rate but with survivors possibly being unaffected by the treatment.

A scaling parameter known as the b value tells us how long it takes for the bacterial colony to shrink by one decimal point. This is expressed as the reciprocal of the microbial inactivation rate. Because relatively low values for *b* were obtained, we may conclude from our analysis that the inactivation process of the bacteria works extremely quickly on the studied materials.

## 3. Materials and Methods

DVB, technical grade (64% o-DVB, m-DVB, p-DVB, and 36% ethylstyrene, as determined by gas chromatography), was purchased from Purolite (Victoria, Romania), and used as received. Benzoyl peroxide (BPO) was obtained from Fluka, and employed as a radical polymerization initiator after recrystallization twice from methanol. Acrylonitrile (AN), purchased from Sigma-Aldrich (Burlington, MA, USA), was distilled at 76–77 °C under a pressure of 101.3 × 10^3^ Pa. 1,2-Diaminoethane (EDA), also purchased from Sigma-Aldrich, was distilled at 118 °C prior to use. Dimethylformamide (DMF), purchased from Sigma-Aldrich, was used as received. HCl and NaOH were purchased from Chemical Company (Iasi, Romania), and used as received. Dibenzylphosphite (Aldrich, Tehn ≥ 85%), diethylphosphite Fluka, Tehn ≥ 90%), 3-pyridinecarboxyaldehyde (Aldrich, 98%), tetrahydrofuran (Sigma-Aldrich, anhydrous, >99.9%) and ethanol p. a. (Chimopar, Bucuresti, Romania) were used as materials for the preparation of polymeric antimicrobial agents.

The antimicrobial activity was tested using standard bacterial strains: Gram-positive *Staphylococcus aureus* (ATCC 25923), and Gram-negative *Escherichia coli* (ATCC 25922), both obtained from Microbiologics Inc. (Saint Cloud, MN, USA). The bacterial cultures were grown on Mueller–Hinton Agar (MHA).

FTIR spectroscopy (KBr pellets) was performed on a JASCO FTIR spectrophotometer (JASCO, Tokyo, Japan) in the range 4000–400 cm^−1^. Products were characterized by thermogravimetric analysis on a TGA/SDTA 851-LF1100-Mettler (Switzerland) device at a temperature ranging from 25 to 800 °C and at a heating rate of 10 °C/min, under nitrogen atmosphere. The elemental composition of the copolymers was evaluated with an Energy-Dispersive X-ray (EDX) analyzer (Octane Elect Super SDD detector, Ametek, Berwyn, PA, USA) equipped on a Verios G4 UC (Thermo Scientific, Brno, Czech Republic) SEM device and Elemental Analyzer UNICUBE—Elementar Analysensysteme GmbH, Langenselbold (Berlin, Germany).

### 3.1. Synthesis of Anion Exchangers with Primary Amino Groups

Briefly, for the preparation of 90 g copolymer with a nominal crosslinking degree of 15%, 21.1 g DVB, 68.9 g AN, 1 wt% BPO, and the porogen agent (calculated for a dilution of 0.15 mL/mL) were used. The synthesis was carried out by suspension polymerization [22], starting with 2 h at 60 °C, followed by 3 h at 70 °C, and then 5 h at 85 °C. The resulting copolymer beads were washed thoroughly with water. The porogen and PAN homopolymer were removed via extraction with methanol and DMF, respectively. Finally, the selected beads (0.3–1.0 mm) were washed with methanol and dried first at room temperature for 24 h, followed by vacuum drying at 50 °C for 48 h. Anion exchangers containing primary amine groups were prepared via the aminolysis–hydrolysis reaction of the nitrile groups with EDA, conducted over 10 h at 118–120 °C, using a molar ratio CN:EDA:H_2_O of 1:10:2. Finally, the beads were filtered and washed with distilled water up to the neutral pH.

The anion exchange capacity was evaluated as follows. Briefly, 15 mL of anion exchanger was measured with a graduated cylinder, placed into a glass column fitted with a borosilicate fritted disk, and thoroughly rinsed with distilled water at neutral pH. A 500 mL aqueous solution of 4 wt% NaOH was then passed through the column for 1 h, followed by extensive rinsing with distilled water to reach neutral pH. Next, 50 mL of 1 N HCl was passed through the column for 30 min, followed by 150 mL of distilled water for 15 min. The effluent was collected in an Erlenmeyer flask and titrated with 1 N NaOH, using an indicator to signal the endpoint by a color change. The anion exchanger was then converted to its free base form by passing another 500 mL of 4% NaOH solution for 1 h, followed by a thorough rinse with distilled water. Finally, the anion exchanger was quantitatively transferred into a graduated cylinder and its volume was measured. The anion exchange capacity per volume (*C_sv_*, meq/mL) was calculated with Equation (3):

(3) $$C_{sv}=\frac{50\times n_{HCl}-V_{NaOH}\times n_{NaOH}}{V}$$

where *V* (mL) is the volume of anion exchanger, while *n_HCl_* and *n_NaOH_* were calculated with Equations (4) and (5):

(4) $$n_{HCl}=\frac{T_{HCl}\times 1000}{E_{HCl}}$$

(5) $$n_{NaOH}=\frac{T_{NaOH}\times 1000}{E_{NaOH}}$$

where *T_HCl_* and *T_NaOH_* are the titers of HCl and NaOH solutions; $E_{HCl}=36.5;E_{NaOH}=40.$

The anion exchanger in swollen state was dried at 105 °C until a constant weight (m, g), and the anion exchange capacity per dry anion exchanger (*C_sg,_* meq/g) was then determined with Equation (6).

(6) $$C_{sg}=\frac{50\times n_{HCl}-V_{NaOH}\times n_{NaOH}}{m}$$

SV3: *C_sv_* = 1.25 meq/mL; *C_sg_* = 2.99 meq/g, (EDX: N = 21.2%, O = 10%) and SV4: *C_sv_* = 1.12 meq/mL; *C_sg_* = 2.42 meq/g, (EDX: N = 25.6%, O = 12.5%).

Before structural and morphological characterization, the anion exchangers were thoroughly washed with anhydrous methanol, air-dried for 24 h at room temperature, and then further dried at 40 °C under reduced pressure for three days.

### 3.2. Chemical Modification of Amino Moieties in AN-15%DVB Copolymers with Aminophosphonate Groups

The functionalization of AN-15%DVB copolymers bearing primary amino groups (SV3 and SV4) was achieved using a “one-pot” K-F reaction with 3-pyridinecarboxyaldehyde and either dibenzylphosphite (resulting in the product Bz-DVB-AN) or diethylphosphite (resulting in the product Et-DVB-AN). The reactions were carried out at a molar ratio of 1:1.3:1.3 for the polymer (AN-DVB):aldehyde:phosphite in tetrahydrofuran at a temperature of 60 °C. After 20 h, the products were filtered, washed with ethanol, and dried at 50 °C for 24 h.

### 3.3. Antibacterian Assay

For the antibacterial investigations, 24 h active cultures of known standard cultures (MediMark Europe Company, Grenoble, France) of Gram-positive bacteria (*S. aureus*, ATCC 25923) and Gram-negative bacteria (*E. coli*, ATCC 25922) were prepared using Mueller–Hinton Agar (MHA) medium. Dilutions were made from active cultures at a level of 1.0 × 10^7^ colony forming units (CFU/mL) on the McFarland scale. A quantity of 29 milliliters of distilled water was added to 0.5 g of each sample (Bz-DVB-AN and Et-DVB-AN). One milliliter of each microbial culture containing 10^7^ CFU/mL (*S. aureus* and *E. coli*) was added to the prepared mixture in turn. The mixtures were then constantly mixed for eighteen hours at room temperature. The decimal dilution method was used to count the number of bacterial cells (colony forming unit—CFU/mL) in each sample at time zero, which was the moment when the sample was combined with the bacterial culture, and subsequently at intervals of 3, 6, 9, 12, and 18 h. Following testing, the samples were sterilized for 30 min at 120 °C and 1 atm.

**Scheme 2:**
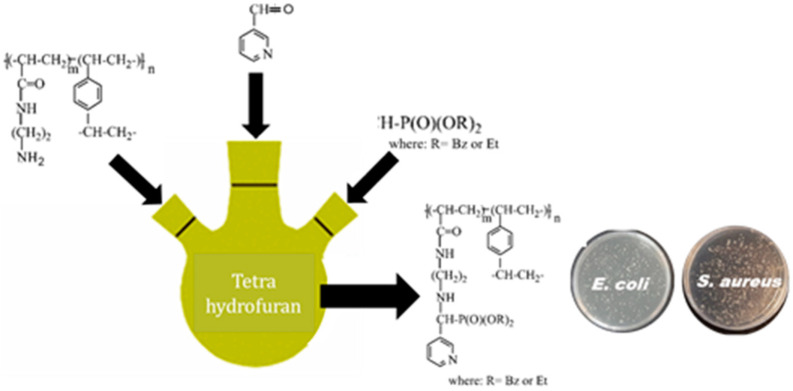
Schematic representation of the synthesis and antimicrobial activity of the functionalized materials.

Scheme 2 presents the synthesis and antimicrobial activity of the functionalized materials.

## 4. Conclusions

We have synthesized two new products that have aminophosphonic acid groups functionalized on a copolymer of acrylonitrile-15% divinylbenzene. The aminophosphonate groups (Bz-DVB-AN, Et-DVB-AN) grafted onto AN-DVB were synthesized following a “one-pot” Kabachnik–Fields reaction. The antibacterial evaluation of Bz-DVB-AN and Et-DVB-AN was tested in vitro against a species of Gram-negative bacteria (*E. coli*) and a type of Gram-positive bacteria (*S. aureus*). Aminophosphonates functionalized on AN-DVB have proven to be new antibacterial agents. It was proven that these compounds exhibit antibacterial activity. In these cases, the cells remaining after 18 h of treatment indicate that they are resistant. The first-order kinetic model and the Weibull model were used to model the kinetic inactivation of *S. aureus* and *E. coli* in Bz-DVB-AN and Et-DVB-AN. After 9 h of interaction, the Gram-positive bacterial load decreased by more than 50%, while the Gram-negative bacteria showed this effect only after 12 h. The samples are slightly more active against Gram-negative bacteria than against Gram-positive bacteria. The effectiveness of the Et-DVB-AN sample on *S. aureus* varies from bacteriostatic (94.8%) to bactericidal (99%). It is observed that the rate of cell reduction for *S. aureus* is quite quick.

## Figures and Tables

**Table 1:** Elemental analysis and semi-quantitative EDX-analysis of SV3 and Bz-DVB-AN copolymers.

Type of Analysis	Wt, %				
	C	N	O	P	H
			SV3		
EDX	68.3	21.7	10	-	-
			Bz-DVB-AN		
Elemental	62.95	14.14	-	-	7.48
EDX	67.1	18	13.7	1.2	-

**Table 2:** Elemental analysis and semi-quantitative EDX-analysis of both SV4 and Et-DVB-AN.

Type of Analysis	Wt, %				
	C	N	O	P	H
			SV4		
EDX	61.7	25.6	12.6	-	-
			Et-DVB-AN		
Elemental	62.97	15.11	-	-	7.5
EDX	74.46	15.55	9.59	0.4	-

**Table 3:** The logarithmic surviving fraction (log(N_t/_N_0_) for both Bz-DVB-AN and Et-DVB-AN vs. the interval time of contact (h).

Time, h	0	3	6	9	12	18
			Log N_t_/N_0_ *			
			*E. coli*			
Bz-DVB-AN	0	−0.0732	−0.1417	−0.2429	−0.3316	−0.5562
Et-DVB-AN	0	−0.1240	−0.3518	−0.4884	−0.5052	−0.8095
			*S. aureus*			
Bz-DVB-AN	0	−0.0777	−0.1505	−0.3410	−0.4134	−0.6090
Et-DVB-AN	0	−0.1560	−0.4179	−0.4227	−1.0087	−1.2839

* N_t_ is the number of germs at time t (h); N_0_ is the initial number of germs.

**Table 4:** First-order kinetic model parameters for *E. Coli* and *S. aureus* reduction by treatment with Bz-DVB-AN and Et-DVB-AN.

Antimicrobial Materials	Kinetic Parameters *	*E. coli*	*S. aureus*
Bz-DVB-AN	R^2^	0.99525	0.99216
	D_T_, h	32.4	28.4
Et-DVB-AN	R^2^	0.98512	0.97158
	D_T_, h	22.6	13.4

* R^2^ is regression coefficient; D_T_ is decimal regression time.

**Table 5:** Weibull model parameters for *E. coli* and *S. aureus* reduction by treatment with Bz-DVB-AN and Et-DVB-AN.

Antimicrobial Materials	Kinetic Parameters *	*E. coli*	*S. aureus*
Bz-DVB-AN	χ^2^	0.00006	0.0014
	b	0.01675	0.02811
	n	1.21009	1.0716
Et-DVB-AN	χ^2^	0.00326	0.002188
	b	0.06697	0.04805
	n	0.85784	1.14772

* b is Weibull scale parameter; n is Weibull dimensionless shape parameter.

## Data Availability

Data are contained within the article.

## Supplementary Materials

The following supporting information can be downloaded at: https://www.mdpi.com/article/10.3390/molecules29246054/s1, Table S1: The total number of germs (CFU/mL) and percentage of bacterial reduction (P, %) at different contact times.
