# Supplementary material for: Chemical Modification of Acrylonitrile-Divinylbenzene Polymer Supports with Aminophosphonate Groups and Their Antibacterial Activity Testing
Source: Molecules. 2024 Dec 23;29(24):6054. doi: 10.3390/molecules29246054 (PMC11677517; doi:10.3390/molecules29246054)
Supplement: Supplementary file 1 [file molecules-29-06054-s001.zip › molecules-3376153-supplementary.pdf]

Chemical Modification of Acrylonitrile-Divinylbenzene Polymer Supports with Aminophosphonate Groups and Their Antibacterial Activity Testing

Ileana Nichita [1], Lavinia Lupa [2], Aurelia Visa [3], Ecaterina-Stela Dragan [4,*], Maria Valentina Dinu [4] and Adriana Popa [3,*]

[1] Faculty of Veterinary Medicine, University of Life Science "King Mihai I", 119 Calea Aradului, 300465 Timisoara, Romania; ileananichita@usab-tm.ro
[2] Faculty of Chemical Engineering, Biotechnology and Environmental Protection, Politehnica University Timișoara, 6 Vasile Parvan Blvd., 300223 Timisoara, Romania; lavinia.lupa@upt.ro
[3] "Coriolan Drăgulescu" Institute of Chemistry, 24 Mihai Viteazul Blv., 300223 Timisoara, Romania; avisa@acad-icht.tm.edu.ro
[4] "Petru Poni" Institute of Macromolecular Chemistry, 41A Aleea Grigore Ghica Vodă, 700487 Iași, Romania; dinu.valentina@icmpp.ro
* Correspondence: sdragan@icmpp.ro (E.-S.D.); apopa_ro@yahoo.com or apopa@acad-icht.tm.edu.ro (A.P.)

Table S1. The total number of germs (CFU/mL) and percentage of bacterial reduction (P, %) at different contact times.

Sample	0 h	3 h		6 h		9 h		12 h		18 h	
	CFU/mL	CFU/mL	P,%	CFU/mL	P,%	CFU/mL	P,%	CFU/mL	P,%	CFU/mL	P,%
Escherichia coli											
BzDVBAN	1.337.216	1.129.816	15.5	964.784	27.8	764.264	42.8	623.126	53.4	371.475	72.2
EtDVBAN	1.269.800	954.262	24.8	564.786	55.5	412.378	67.5	396.762	68.7	196.850	84.4
Staphylococcus aureus											
BzDVBAN	2.952.770	2468942	16.8	2087562	29.3	1.346.463	54.4	1.139.769	61.4	726.381	75.4
EtDVBAN	2.572.250	1.795.893	30.1	982.553	61.8	971.692	62.2	252.080	90.2	133.757	94.8
